# HyperGraphs.jl: representing higher-order relationships in Julia

**DOI:** 10.1093/bioinformatics/btac347

**Published:** 2022-06-08

**Authors:** Léo P M Diaz, Michael P H Stumpf

**Affiliations:** Melbourne Integrative Genomics and School of Mathematics and Statistics, University of Melbourne, Melbourne, Parkville, VIC 3010, Australia; Melbourne Integrative Genomics and School of Mathematics and Statistics, University of Melbourne, Melbourne, Parkville, VIC 3010, Australia; School of BioSciences, University of Melbourne, Melbourne, Parkville, VIC 3010, Australia

## Abstract

**Summary:**

HyperGraphs.jl is a Julia package that implements hypergraphs. These are a generalization of graphs that allow us to represent *n*-ary relationships and not just binary, pairwise relationships. High-order interactions are commonplace in biological systems and are of critical importance to their dynamics; hypergraphs thus offer a natural way to accurately describe and model these systems.

**Availability and implementation:**

HyperGraphs.jl is freely available under the MIT license. Source code and documentation can be found at https://github.com/lpmdiaz/HyperGraphs.jl.

**Supplementary information:**

Supplementary data are available at *Bioinformatics* online.

## 1 Introduction

Graphs offer flexible ways to represent many biological systems. They are a well-established tool, and graph-based modeling formalisms such as chemical reaction networks and Petri nets have become widely used frameworks to describe and model such systems. Here, vertices are often taken to represent genes or gene products, with edges describing their interactions. Graphs are, however, constrained to only representing pairwise interactions. This is a major limitation as not all complex processes can be understood solely in terms of pairwise relationships, and higher-order interactions are increasingly being recognized as a natural and often essential property of many dynamical systems (Battiston *et al.*, 2021; Lambiotte *et al.*, 2019).

Hypergraphs are mathematical objects that can naturally represent high-order relationships, making them obvious candidates to accurately describe biological systems (Klamt *et al.*, 2009). Their adoption as a modeling framework has, however, been slow. One reason for this may have been a lack of computational implementations (see Supplementary Material). Our package, HyperGraphs.jl, fills this gap within the Julia package ecosystem.

## 2 Methods and features

### 2.1 Hypergraphs and dynamical systems

**Fig. 1.**
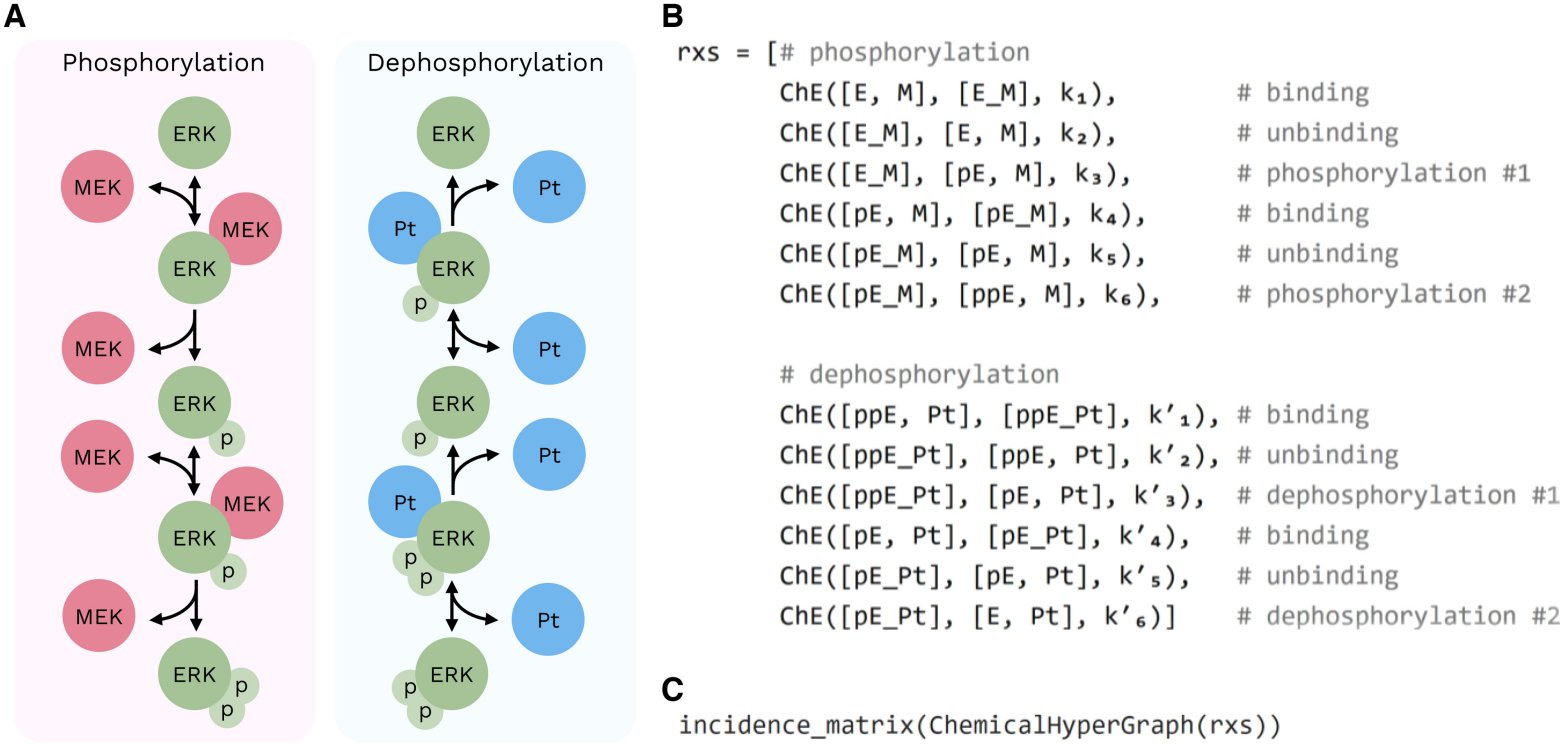
Usage example of HyperGraphs.jl. Model of the MEK/ERK signaling cascade adapted from Filippi *et al.* (2016) (**A**) and its definition as a chemical hypergraph (**B**), where ChE stands for chemical hyperedge, and species (with M representing MEK; E, ERK; Pt, phosphatase; and p, a phosphor group) are implemented as symbolic variables via Symbolics.jl (Gowda *et al.*, 2021). Note that if left unspecified, vertex multiplicity (encoding the stoichiometric coefficient of each species in a chemical hyperedge) defaults to 1. (**C**) Code returning the incidence matrix where entries collect the sum of vertex multiplicities, which corresponds to the stoichiometric matrix of that reaction system. See Supplementary Material for the full example

Hypergraphs are defined as a pair (*V*, *E*) with *V* a set of *vertices* and *E* a set of *hyperedges* constructed from (multi)sets of elements in *V* (in a multiset each element can occur more than once). This formalism offers flexible descriptions of dynamical systems: vertices represent objects of interest and are endowed with a dynamical variable tracking a quantity of interest over time, and hyperedges describe relationships between any number of objects; see for example Figure 1A.

### 2.2 Biological relevance: chemical hypergraphs

Chemical hypergraphs (Jost and Mulas, 2019) are a representation of chemical reaction networks where vertices represent chemical species and hyperedges describe reactions. HyperGraphs.jl provides an implementation of chemical hypergraphs, understood as a special case of hypergraph. Chemical hypergraphs are *oriented* hypergraphs where each hyperedge has a source and target set corresponding to reactants and products, respectively. In addition, each hyperedge is annotated with a real number encoding the reaction rate and each vertex in a hyperedge is annotated with a positive integer representing its stoichiometric coefficient in the reaction (Fig. 1B).

This description can then be linked to the system’s dynamics over time by defining a notion of kinetics on the chemical hypergraph (e.g. mass-action kinetics), as is routinely done with chemical reaction networks.

### 2.3 Additional features

We implement important graph-theoretic concepts needed for the representation, description and analysis of hypergraphs and their components. This includes hyperedge and hypergraph properties and hypergraph matrices, including, for example, the incidence matrices of unoriented and oriented hypergraphs. Importantly, this allows us to ground general biochemical models in a graph-theoretic framework. This shift in perspective has immediate implications: for instance, the time evolution of a chemical reaction network may be written as a matrix product of certain matrices arising from that representation (Gatermann and Huber, 2002). This approach has important precedents (Othmer, 1979) but is rarely made explicit. The relevant matrix types (as well as other graph-theoretic concepts) are directly accessible when casting models of biochemical systems as hypergraphs, as illustrated in Figure 1C.

HyperGraphs.jl also provides functions to modify hypergraphs, making it straightforward to manipulate and edit mathematical models. This gives us a way to explore model space by dynamically altering the model’s structure.

## 3 Conclusion

HyperGraphs.jl provides a general framework to intuitively model systems with high-order interactions. Chemical hypergraphs are specifically designed to represent reactions and as such are appropriate for describing reaction-based biological systems. Hypergraphs are however more general and able to represent a wide range of systems beyond biological. Mathematical models cast as hypergraphs are naturally rooted in graph theory. In this way, understanding existing modeling frameworks through the lens of graph theory enables hypergraphs to act as a bridge between different formalisms. A clear benefit stems, for example, from the equivalence between chemical reaction networks and types of Petri nets—see e.g. Baez and Pollard (2017)—as results in one formalism may be transferred to the other via the shared language of hypergraphs. In addition, HyperGraphs.jl easily interoperates with other Julia packages. Hypergraphs can, for instance, be used in a symbolic programming context. In this way, hyperedges describe how symbolic variables (represented by vertices, as done in Fig. 1B) interact, yielding symbolic equations that can subsequently be solved via the DifferentialEquations.jl package ecosystem (Rackauckas and Nie, 2017).

HyperGraphs.jl implements graph-theoretic concepts that can exploit the advantages of hypergraphs in representing mathematical models. Our package thus provides practical tools to naturally model the high-order interactions crucial to understanding complex system dynamics.

## Funding

This work was supported by the Melbourne Research Scholarship from the University of Melbourne (LPMD).

*Conflict of Interest*: The authors declare that they have no conflict of interest.

## Supplementary Material

btac347_Supplementary_Data
