# Supplementary material for: HyperGraphs.jl: representing higher-order relationships in Julia
Source: Bioinformatics. 2022 Jun 8;38(14):3660–1. doi: 10.1093/bioinformatics/btac347 (PMC9326852; doi:10.1093/bioinformatics/btac347)
Supplement: btac347_Supplementary_Data [file btac347_supplementary_data.pdf]

Supplementary Information

HyperGraphs.jl – representing high-order relationships in Julia

Léo P.M. Diaz [1] and Michael P.H. Stumpf [1,2]

[1]Melbourne Integrative Genomics and School of Mathematics and Statistics, University of Melbourne, Melbourne, Parkville, 3010, VIC, Australia and [2]School of BioSciences, University of Melbourne, Melbourne, Parkville, 3010, VIC, Australia

A Examples

At the time of writing, these examples run with Julia v1.7.2, ModelingToolkit.jl v8.5.5, OrdinaryDiffEq.jl v6.7.1, and Plots.jl v1.27.3. An archived version of HyperGraphs.jl that runs this code can be found at Zenodo. Both examples are also available as an executable Jupyter notebook.

A.1 Standalone use of HyperGraphs.jl

This example demonstrates how to use HyperGraphs.jl on its own.

```julia
using HyperGraphs
const ChE = ChemicalHyperEdge

k₁, k₂, k₃, k₄, k₅, k₆ = :k₁, :k₂, :k₃, :k₄, :k₅, :k₆
k′₁, k′₂, k′₃, k′₄, k′₅, k′₆ = :k′₁, :k′₂, :k′₃, :k′₄, :k′₅, :k′₆
E, M, pE, ppE, Pt = :E, :M, :pE, :ppE, :Pt
E_M, pE_M, pE_Pt, ppE_Pt = :E_M, :pE_M, :pE_Pt, :ppE_Pt

rxs1 = [# phosphorylation reactions
        ChE([E, M], [E_M], k₁),         # binding
        ChE([E_M], [E, M], k₂),         # unbinding
        ChE([E_M], [pE, M], k₃),        # phosphorylation #1
        ChE([pE, M], [pE_M], k₄),       # binding
        ChE([pE_M], [pE, M], k₅),       # unbinding
        ChE([pE_M], [ppE, M], k₆),      # phosphorylation #2

        # dephosphorylation reactions
        ChE([ppE, Pt], [ppE_Pt], k′₁), # binding
        ChE([ppE_Pt], [ppE, Pt], k′₂), # unbinding
        ChE([ppE_Pt], [pE, Pt], k′₃),  # dephosphorylation #1
        ChE([pE, Pt], [pE_Pt], k′₄),   # binding
        ChE([pE_Pt], [pE, Pt], k′₅),   # unbinding
        ChE([pE_Pt], [E, Pt], k′₆)]    # dephosphorylation #2

X1 = ChemicalHyperGraph(rxs1)

(nv(X1), nhe(X1))

incidence_matrix(X1)
```

A.2 Using HyperGraphs.jl together with other Julia packages

This example shows interoperability between HyperGraphs.jl and other Julia packages (here, ModelingToolkit.jl, OrdinaryDiffEq.jl, and Plots.jl).

```julia
using HyperGraphs, ModelingToolkit, OrdinaryDiffEq, Plots
const ChE = ChemicalHyperEdge

@parameters t k₁ k₂ k₃ k₄ k₅ k₆ k′₁ k′₂ k′₃ k′₄ k′₅ k′₆
@variables E(t) M(t) pE(t) ppE(t) Pt(t) E_M(t) pE_M(t) pE_Pt(t) ppE_Pt(t)

rxs2 = [# phosphorylation reactions
        ChE([E, M], [E_M], k₁),          # binding
        ChE([E_M], [E, M], k₂),          # unbinding
        ChE([E_M], [pE, M], k₃),         # phosphorylation #1
        ChE([pE, M], [pE_M], k₄),        # binding
        ChE([pE_M], [pE, M], k₅),        # unbinding
        ChE([pE_M], [ppE, M], k₆),       # phosphorylation #2

        # dephosphorylation reactions
        ChE([ppE, Pt], [ppE_Pt], k′₁), # binding
        ChE([ppE_Pt], [ppE, Pt], k′₂), # unbinding
        ChE([ppE_Pt], [pE, Pt], k′₃),  # dephosphorylation #1
        ChE([pE, Pt], [pE_Pt], k′₄),   # binding
        ChE([pE_Pt], [pE, Pt], k′₅),   # unbinding
        ChE([pE_Pt], [E, Pt], k′₆)]    # dephosphorylation #2

X2 = ChemicalHyperGraph(rxs2)

mass_action(e::ChemicalHyperEdge) = rate(e) * prod(src(e) .^ src_stoich(e))
mass_action(x::ChemicalHyperGraph) = mass_action.(hyperedges(x))

lhss = [Differential(t)(v) for v in vertices(X2)]
rhss = incidence_matrix(X2) * mass_action(X2)
eqs  = Equation.(lhss, rhss)

@named sys = ODESystem(eqs, t, vertices(X2), weights(X2))

u0 = [E => 2000, M => 636, E_M => 0, pE => 0, pE_M => 0,
      ppE => 188, Pt => 1000, ppE_Pt => 0, pE_Pt => 0]

p = [k₁ => 0.5, k₂ => 0.5, k₃ => 450, k₄ => 249.5, k₅ => 0.5, k₆ => 5,
     k′₁ => 499.5, k′₂ => 49999.5, k′₃ => 50, k′₄ => 5, k′₅ => 500, k′₆ => 50000]

tspan = (0.0, 0.1)
prob = ODEProblem(sys, u0, tspan, p)
sol = solve(prob, Tsit5())
plot(sol)
```

B Comparison of HyperGraphs.jl against other resources

There are few computational implementations of resources allowing to describe high-order interactions; the table below lists those known to us.

resource	link	language
HyperGraphs.jl	https://github.com/lpmdiaz/HyperGraphs.jl	Julia
HyperNetX	https://github.com/pnnl/HyperNetX	Python
SimpleHypergraphs.jl	https://github.com/pszufe/SimpleHypergraphs.jl	Julia

Hypergraphs give us more flexibility than graphs to represent complex systems: unlike the latter, they naturally allow to represent high-order relationships. We are interested in using hypergraphs as means to an end: they allow us to describe a system's structure (inasmuch that hyperedges describe how objects of interest interact), which we then want to use to either simulate the system or analyse it further. We are thus not interested in computational implementations that only allow to build hypergraphs; we need code that can be re-purposed, and that can be used with existing packages or with custom-built frameworks. HyperGraphs.jl is designed to specifically allow for this. This means that HyperGraphs.jl, despite also implementing high-order interactions, differs from the other resources in the table above in two main ways:

1 Generality & abstraction. The direction taken in HyperGraphs.jl is to keep the code as general as possible. This focus on generality in turn provides flexibility: by implementing building blocks in an abstract way, they can be used in a wide variety of cases. For instance, functions returning e.g. the vertices or the hyperedges of a hypergraph work on any type of hypergraph: they work for the currently implemented chemical hypergraphs, but will naturally extend to any other type of hypergraph (e.g. Petri nets) implemented in the future. This implies that our code is not application-specific and can be used for a variety of (potentially custom) use cases. SimpleHypergraphs.jl and HyperNetX have not been designed with a focus on generality. The latter is also more limited than the former: in both SimpleHypergraphs.jl and HyperGraphs.jl, vertices may be of any type (thus allowing users to use custom types and represent relations between those), while in HyperNetX vertices may be objects that are not numbers but cannot be e.g. lists.

2 Intuitiveness & efficiency. HyperGraphs.jl is written in the Julia language, meaning it benefits from Julia's advantages over other languages. Some of these are: (i) the intuitiveness of Julia, as code is highly readable even for non-Julia users; (ii) the ability for code written in Julia to compose with other packages (meaning that functions from one package can often be used by another package natively); and (iii) the possibility to use multiple typing to overload functions and thus extend existing functionalities to new cases, which is highly appropriate for our aim of code re-purposing. Comparing HyperGraphs.jl to the other resources, SimpleHypergraphs.jl is arguably less intuitive to use – constructors appear more opaque and less obvious than those in HyperGraph.jl – and HyperNetX does not benefit from the composability inherent to Julia, which is what makes the language so powerful and attractive.

Additionally, SimpleHypergraphs.jl seems to use the hypergraph's incidence matrix as its underlying representation; this may be problematic since two distinct hypergraphs may share the same incidence matrix despite not having identical hyperedges, due to the incidence matrix collecting *sums* of incidence multiplicities which thus discards some information.

Together, the specific focus of HyperGraphs.jl on generality and it being written in the Julia language means that the functionalities implemented by this package are not directly comparable to those implemented by other resources providing implementations of high-order relationships.
